# Multispecimen Genomic Profiling Reveals Inter-Specimen Heterogeneity and Supports Ascites as a Complementary Biospecimen in Pseudomyxoma Peritonei

**DOI:** 10.3390/diagnostics16213463

**Published:** 2026-10-23

**Authors:** Shi Yan, Huanhuan Liu, Yiming Kang, Chong Wang, Pu Zhang, Haipeng Zhou, Zijie Huang, Guangjun Shi, Lubiao An, Huidan Zhang, Hongyu Zhao, Hongbin Xu, Ruiqing Ma

**Affiliations:** 1 Department of Myxoma, Aerospace Center Hospital, Beijing 100049, China; 2 Suzhou Geno-Truth Diagnostics Co., Ltd., Suzhou 215128, China; 3 Beijing Champion Medical Co., Ltd., Beijing 102206, China; 4 School of Basic Medicine, Capital Medical University, Beijing 100069, China

**Keywords:** pseudomyxoma peritonei, ascites fluid, concordance, KRAS subgroups, actionable alterations

## Abstract

**Background**: Pseudomyxoma peritonei (PMP) is a rare peritoneal malignancy with limited genomic data across different biospecimen types. The extent to which ascites and plasma reflect tissue-detected genomic alterations remains incompletely characterized. This study compared genomic profiles across tumor tissue, ascites, and plasma and explored KRAS-defined molecular features in PMP. **Methods**: Sixty patients with pathologically confirmed PMP were retrospectively included. Next-generation sequencing (NGS) data were available from tumor tissue in 57 patients, ascites in 40, and plasma in 28, with overlapping specimen availability. Cross-specimen genomic concordance was evaluated in 37 patients with matched tissue–ascites samples, including 28 patients with matched tissue, ascites, and plasma. Exploratory analyses were performed to characterize genomic and pathway-level features across KRAS-defined subgroups. **Results**: In tumor tissue, KRAS (66.7%), GNAS (31.6%), and TP53 (21.1%) were the most frequently altered genes. Among 28 patients with matched tissue, ascites, and plasma, patient-level concordance was 35.7%. At the mutation level, ascites showed higher tissue-referenced positive percentage agreement (PPA) than plasma (93.1% vs. 36.2%). In the expanded cohort of 37 matched tissue–ascites pairs, ascites achieved a tissue-referenced PPA of 94.2% and revealed additional alterations not detected in the corresponding tissue samples. Exploratory KRAS mutation stratification identified G12D (35.0%), G12X (32.5%), and KRAS-wild-type (wt) (17.5%) tumors as the predominant subgroups. KRAS-mutant tumors harbored more detected genomic alterations and potentially clinically actionable alterations than KRAS-wt tumors. Wnt and PI3K pathway alterations were more commonly observed in the G12D and G12X subgroups, respectively, whereas canonical oncogenic pathway alterations were less frequent in KRAS-wt tumors. **Conclusions**: PMP exhibits substantial genomic heterogeneity across patients and biospecimen types. Ascites cfDNA showed high concordance with tissue and captured additional genomic alterations, suggesting that ascitic fluid may serve as a complementary source for molecular profiling in PMP. KRAS-defined subgroups exhibited distinct genomic characteristics, although their clinical relevance requires further validation in larger, well-characterized cohorts.

## 1. Introduction

Pseudomyxoma peritonei (PMP) is a rare clinicopathological condition characterized by the progressive intraperitoneal dissemination of mucinous neoplasia, most commonly originating from an appendiceal mucinous tumor [1,2,3]. PMP encompasses a heterogeneous disease spectrum with considerable variation in histopathological grade, tumor distribution, clinical behavior, and prognosis. Cytoreductive surgery (CRS) combined with hyperthermic intraperitoneal chemotherapy (HIPEC) is the standard treatment for eligible patients [4]. However, recurrence remains common even after complete cytoreduction, systemic therapies have shown limited efficacy, and patients ineligible for CRS-HIPEC have few effective treatment options [5]. These limitations highlight the need for a better understanding of the molecular heterogeneity of PMP and the identification of clinically relevant genomic alterations.

Next-generation sequencing (NGS) has expanded our understanding of the molecular landscape of PMP, but comprehensive molecular characterization remains challenging because of disease-specific pathological and sampling features. PMP specimens often contain abundant extracellular mucin and sparse tumor cells, with tumor cellularity frequently below 5%, which may limit the sensitivity of tissue-based genomic profiling [6,7]. Plasma-based detection of tumor-derived alterations has also shown limited sensitivity in PMP and other malignancies predominantly confined to the peritoneal cavity [8,9]. Ascitic fluid, when available, is in direct contact with disseminated peritoneal lesions and may contain tumor-derived molecular material from multiple disease sites. It may therefore serve as a complementary biospecimen for genomic profiling in PMP. However, systematic comparisons of genomic alterations across tumor tissue, ascitic fluid, and plasma remain limited.

KRAS mutations represent one of the most prevalent oncogenic alterations in PMP, with reported frequencies varying considerably across studies [1]. Emerging evidence from other malignancies indicates that individual KRAS variants may differ in downstream signaling, co-alteration patterns, and biological behavior [10,11]. Whether similar variant-specific molecular heterogeneity exists in PMP remains poorly characterized. Moreover, given the substantial clinicopathological heterogeneity of PMP and the limited size of existing molecular cohorts, the clinical relevance of KRAS-defined subgroups remains to be established.

In this study, we performed multispecimen genomic profiling of PMP using tumor tissue, ascitic fluid, and plasma to characterize inter-specimen molecular heterogeneity and evaluate the extent to which ascitic fluid captures tumor-associated genomic alterations. We further explored the genomic, pathway-level, and clinical characteristics of KRAS-defined subgroups to provide a more comprehensive characterization of the molecular diversity of PMP.

## 2. Material and Methods

### 2.1. Study Design and Patient Enrollment

**Figure 1:**
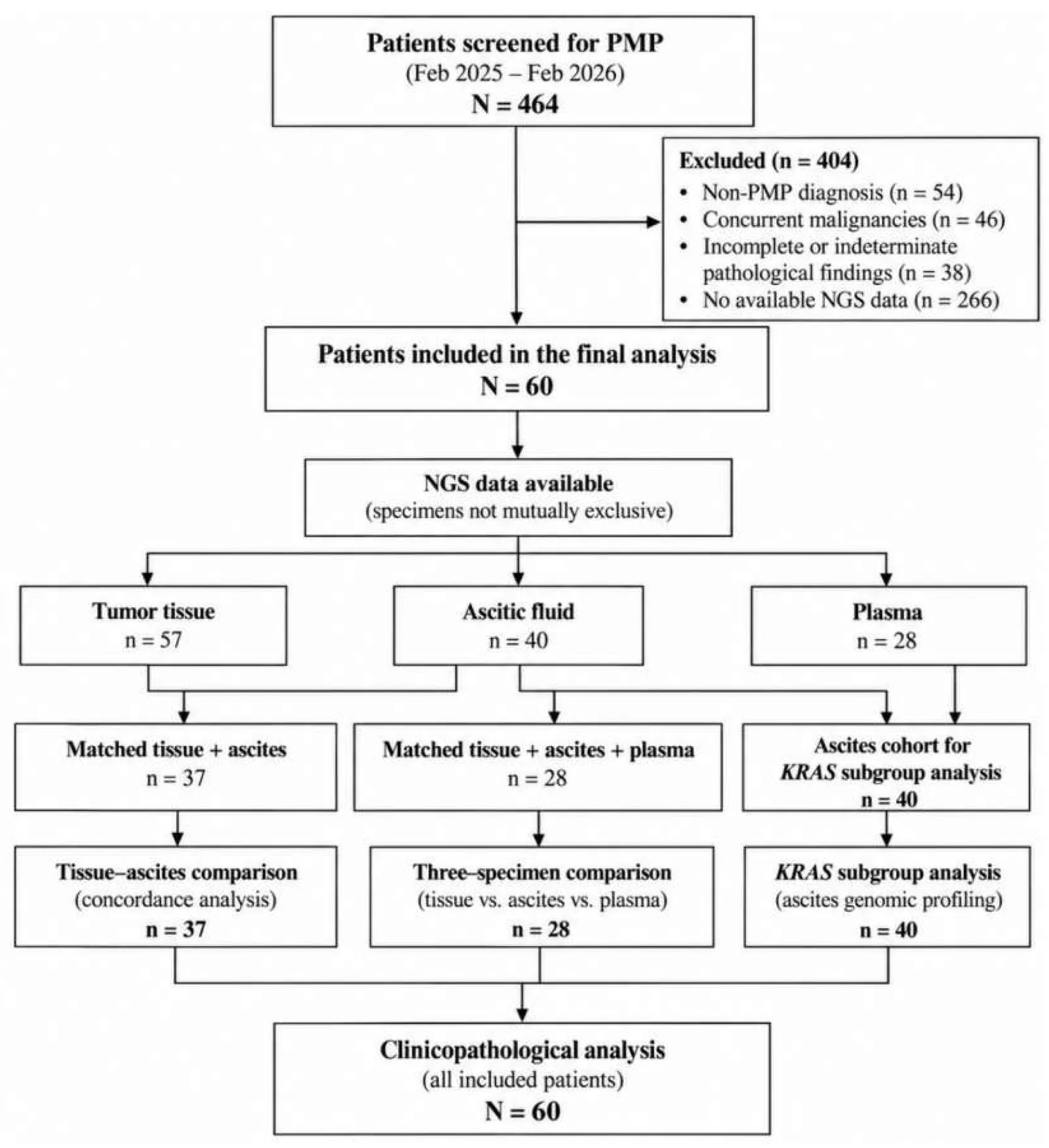
Study flowchart and analytical cohorts.

This retrospective study screened 464 consecutive patients with a clinical or pathological diagnosis of PMP at Aerospace Center Hospital between February 2025 and February 2026. PMP was defined as histopathologically confirmed intraperitoneal dissemination of mucinous neoplasia, characterized by mucinous peritoneal deposits with variable amounts of neoplastic epithelium. The presence of free mucinous ascites was not required for the diagnosis of PMP. After applying the predefined inclusion and exclusion criteria, 60 patients were included in the final analysis (Figure 1). Eligible patients were required to have histopathologically confirmed PMP and at least one tumor-associated specimen with available NGS data. Patients with concurrent malignancies, non-PMP diagnoses, incomplete or indeterminate pathological findings, and unavailable NGS data were excluded. The primary tumor site and clinicopathological characteristics were determined from available surgical and pathological records. Histopathological classification was based on the Peritoneal Surface Oncology Group International (PSOGI) classification system. Clinical stage was extracted from the original medical records. Because the cohort included patients with different primary tumor sites, the recorded stages were not assumed to follow a uniform staging system and were used for descriptive purposes only. Cytoreduction outcomes were assessed using the completeness of cytoreduction (CC) score. For the exploratory KRAS subgroup analysis, complete cytoreduction was defined as CC0 and incomplete cytoreduction as CC1–CC3.

Among the 60 included patients, NGS data were available from tumor tissue in 57 patients, ascitic fluid in 40 patients, and plasma in 28 patients. Because specimen availability varied among patients, these groups were not mutually exclusive. The 57 patients with tissue NGS data were used to characterize the tissue-based genomic landscape, whereas the 40 patients with ascites NGS data constituted the cohort for exploratory KRAS subgroup analyses. For cross-specimen comparisons, 37 patients had matched tumor tissue and ascitic fluid samples. Of these, 28 also had matched plasma samples and therefore constituted the three-specimen subset used for direct comparison of genomic alterations across tumor tissue, ascites, and plasma. Accordingly, the 28-patient three-specimen cohort was nested within the 37-patient tissue–ascites cohort rather than representing an independent patient group. The study was approved by the Ethics Committee of Aerospace Center Hospital and conducted in accordance with the Declaration of Helsinki (1964) and its subsequent amendments.

### 2.2. Sample Collection and Processing

For patients with paired specimens, peripheral blood was collected preoperatively on the day of surgery, whereas tumor tissue and ascites were collected intraoperatively before HIPEC. Plasma and leukocytes were isolated from peripheral blood. Samples were stored at 4 °C after collection and processed on the same day. Ascites samples were aliquoted into 50 mL tubes and centrifuged at 1600× *g* for 10 min. The supernatant was transferred to 5 mL tubes and centrifuged at 12,000× *g* for 10 min. It was then transferred to fresh 5 mL tubes and centrifuged again at 12,000× *g* for 10 min. The final supernatant was collected for cell-free DNA (cfDNA) extraction.

### 2.3. Library Preparation and Next-Generation Sequencing

DNA (DNA) was extracted using commercial kits according to the manufacturers’ instructions. Genomic DNA (gDNA) from fresh tissue and peripheral blood leukocytes was isolated using the DNA Kit (HZY11; Suzhou Geno-Truth Diagnostics Co., Ltd., Suzhou, China), whereas cfDNA from plasma and ascites supernatant was extracted using the Circulating DNA Extraction Kit (HZY12; Suzhou Geno-Truth Diagnostics Co., Ltd., Suzhou, China). For ascites supernatant, lysis buffer, protease, and carrier RNA were added in amounts appropriate to the sample volume. Samples were mixed and incubated at 56 °C for 30 min. Binding buffer was then added, followed by incubation on ice for 5 min. The lysate was passed through the extraction column to bind DNA, which was subsequently washed and eluted. The concentration of cfDNA extracted from plasma and ascites supernatant was measured using a Qubit 4 fluorometer (Thermo Scientific, Waltham, MA, USA), and fragment size distribution was assessed using a LabChip GX Touch Nucleic Acid Analyzer (PerkinElmer, Waltham, MA, USA). A total cfDNA yield of at least 20 ng and a predominant fragment peak between 150 and 200 bp were required before library preparation. Sequencing libraries were prepared using the Fast Plus DNA Library Preparation Kit (ABclonal, Woburn, MA, USA; Cat. No. RK20208).

Targeted sequencing was performed using the testing company’s existing hybrid-capture pan-cancer panels covering 1299 or 272 cancer-related genes. Tissue samples were analyzed using the 1299-gene panel. Tissue sequencing identified mutations concentrated in a relatively small set of genes, which informed the selection of the 272-gene panel for plasma and ascites supernatant samples. Sequencing was performed on a DNBSEQ-T7 instrument (MGI, Wuhan, China), with sequencing depth requirements of >1000× for tissue and >10,000× for plasma and ascites supernatant.

Reads were aligned to the human reference genome GRCh37/hg19 using Burrows–Wheeler Aligner (BWA, version 0.7.19). Single-nucleotide variants (SNVs) and insertions/deletions (indels) were called using Mutect2 (GATK, version 4.6.1.0) and FreeBayes (version 1.3.10). For tissue samples, SNVs and indels required a variant allele frequency (VAF) of ≥1% and at least 30 supporting reads. A minimum VAF threshold of 0.1% was applied to plasma and ascites supernatant samples. Matched leukocyte sequencing served as a normal control to distinguish somatic alterations from germline variants and potential clonal hematopoiesis-related variants. Leukocyte sequencing results were evaluated together with annotations from an in-house proprietary database maintained by Geno-Truth Diagnostics to filter germline and suspected clonal hematopoiesis-related variants. Low-quality calls and potential sequencing artifacts were also removed. Copy-number variants (CNVs) were inferred separately using CNVkit (version 0.9.11), with read-depth normalization against a pooled reference.

### 2.4. Define Clinically Actionable Alterations

Clinically actionable alterations were annotated according to the OncoKB evidence framework. Level A (OncoKB Levels 1–2) comprised biomarkers predictive of response to FDA-approved therapies or recommended by clinical guidelines, whereas Level B (OncoKB Levels 3–4) included biomarkers supported by compelling clinical or strong biological evidence of actionability [12]. On-label therapies referred to drugs approved for PMP, whereas off-label therapies were approved for other tumor types but not for PMP. All annotations were based on the OncoKB database (accessed October 2025), and only somatic alterations were evaluated.

### 2.5. Concordance Analysis

Concordance was assessed among matched tissue, ascites, and plasma specimens and between paired tissue and ascites specimens. Analyses were restricted to genomic loci covered by both sequencing panels to ensure comparability across specimen types. The genes corresponding to these shared targeted regions are listed in Supplementary Table S1. Loci covered by only one panel were excluded from the comparisons.

At the patient level, concordance was defined as the detection at least one genomic alteration shared across all specimen types or no detectable alterations in any specimen. It was calculated as (U + D)/N, where U denotes the number of patients with at least one shared alteration across all specimen types, D the number with negative results in all specimens, and N the number with matched results for all specimen types. This measure did not require identical mutation profiles across specimens [13].

At the gene level, paired tissue and ascites specimens were classified as positive or negative for each gene according to whether at least one alteration meeting the filtering criteria was detected. Agreement required matching status, but not necessarily the same alteration. For each gene, V denoted all patients with paired results, and S and C denoted those positive in tissue and ascites, respectively. The complementary sets S^c^ = V\S and C^c^ = V\C denoted patients with negative results. With tissue as the reference, positive percent agreement (PPA) was calculated as |S ∩ C|/|S|, negative percent agreement (NPA) as |S^c^ ∩ C^c^|/|S^c^|, and overall percent agreement (OPA) as (|S ∩ C| + |S^c^ ∩ C^c^|)/|V|. These measures represented agreement among tissue-positive, tissue-negative, and all paired patients, respectively [14].

At the mutation level, matching required the same individual alteration; different alterations within the same gene were treated as distinct events. Overlap across all specimen types was visualized using an UpSet plot, with each intersection bar indicating the number of alterations in the corresponding specimen combination. Mutation-level PPA was calculated separately for ascites and plasma as the proportion of tissue-detected alterations also identified in the corresponding liquid specimen. NPA and OPA were calculated only for the gene-level tissue–ascites comparison. All agreement measures were expressed as percentages.

### 2.6. Analyzing Mutational Signatures

Mutational signature analysis was performed on 57 samples with detected mutations using the R package MutationalPatterns (version 3.16.0). A total of 235 SNVs were included in the analysis. SNVs were classified into 96 trinucleotide substitution categories according to the substitution type and the immediately flanking 5′ and 3′ nucleotides using the GRCh37 reference genome. The resulting sample-by-mutation-category matrix was used for mutational profile analysis. Non-negative matrix factorization (NMF) was applied to extract mutational profiles from the mutation count matrix. The extracted profiles were compared with the COSMIC v3.4 single-base substitution (SBS) reference signatures defined for GRCh37 using cosine similarity. Profiles were classified as unmatched if their maximum cosine similarity to the reference signatures was below 0.80. One such profile was designated Signature A for descriptive purposes. This designation indicated that the profile did not meet the predefined similarity threshold for assignment to the reference signatures examined and did not establish a novel or PMP-specific mutational process. Given the limited number of SNVs and the restricted genomic coverage of targeted sequencing, the mutational signature analysis was considered exploratory.

### 2.7. Statistical Analysis and Software

All statistical analyses were performed using R software (version 4.4.3). Categorical variables were compared using Fisher’s exact test. For continuous variables, normality was assessed using the Shapiro–Wilk test. Comparisons between two groups were performed using Student’s *t*-test or the Mann–Whitney U test, as appropriate, whereas comparisons among three or more groups were performed using one-way analysis of variance (ANOVA) or the Kruskal–Wallis test, as appropriate. Multiple testing was controlled using the Benjamini–Hochberg false discovery rate (FDR) method. All statistical tests were two-sided, and FDR-adjusted *p* values < 0.05 were considered statistically significant unless otherwise specified. Analyses were conducted at the patient level unless otherwise indicated. Data visualization was performed using the R package ggplot2 (version 4.0.3). ChatGPT (OpenAI) was used to assist with English language editing, sentence restructuring, and improving the clarity and organization of selected sections of the manuscript. All AI-assisted text was reviewed and revised by the authors, who retained full responsibility for the scientific content and interpretation of the findings.

## 3. Result

### 3.1. Baseline Clinicopathological Characteristics

A total of 60 patients were included in the final analysis. Baseline demographic and clinical characteristics are summarized in Table 1 and Supplementary Table S1. The median age was 57 years (range: 50–79 years), and females predominated (male-to-female ratio: 1:1.9). The primary tumor site was identified in 47 patients (78.3%). Among these patients, 41 (87.2%) had an appendiceal primary tumor, whereas the remaining cases originated from the ovary (*n* = 2), gastrointestinal tract of unspecified origin (*n* = 2), colon (*n* = 1), or urachus (*n* = 1). The cohort included both treatment-naïve and previously treated patients. Overall, 27 patients (45.0%) had no prior treatment, whereas 32 (53.3%) had undergone prior surgery at an outside hospital, seven (11.7%) had received prior chemotherapy, and six (10.0%) had previously undergone HIPEC; these treatment categories were not mutually exclusive. Stage III was recorded in 65% of patients in the original medical records. A total of 86% underwent CRS combined with HIPEC at our center. Among patients with available cytoreduction data, 28% had achieved complete or near-complete cytoreduction (CC0/1). High-grade peritoneal pseudomyxoma was observed in 64.6% of cases, and mucinous ascites was documented in 63.1% of cases. Tumor cellularity was generally low, with 77.6% of evaluable tissue specimens containing ≤20% tumor cells.

### 3.2. Genomic Landscape of Pmp

**Figure 2:**
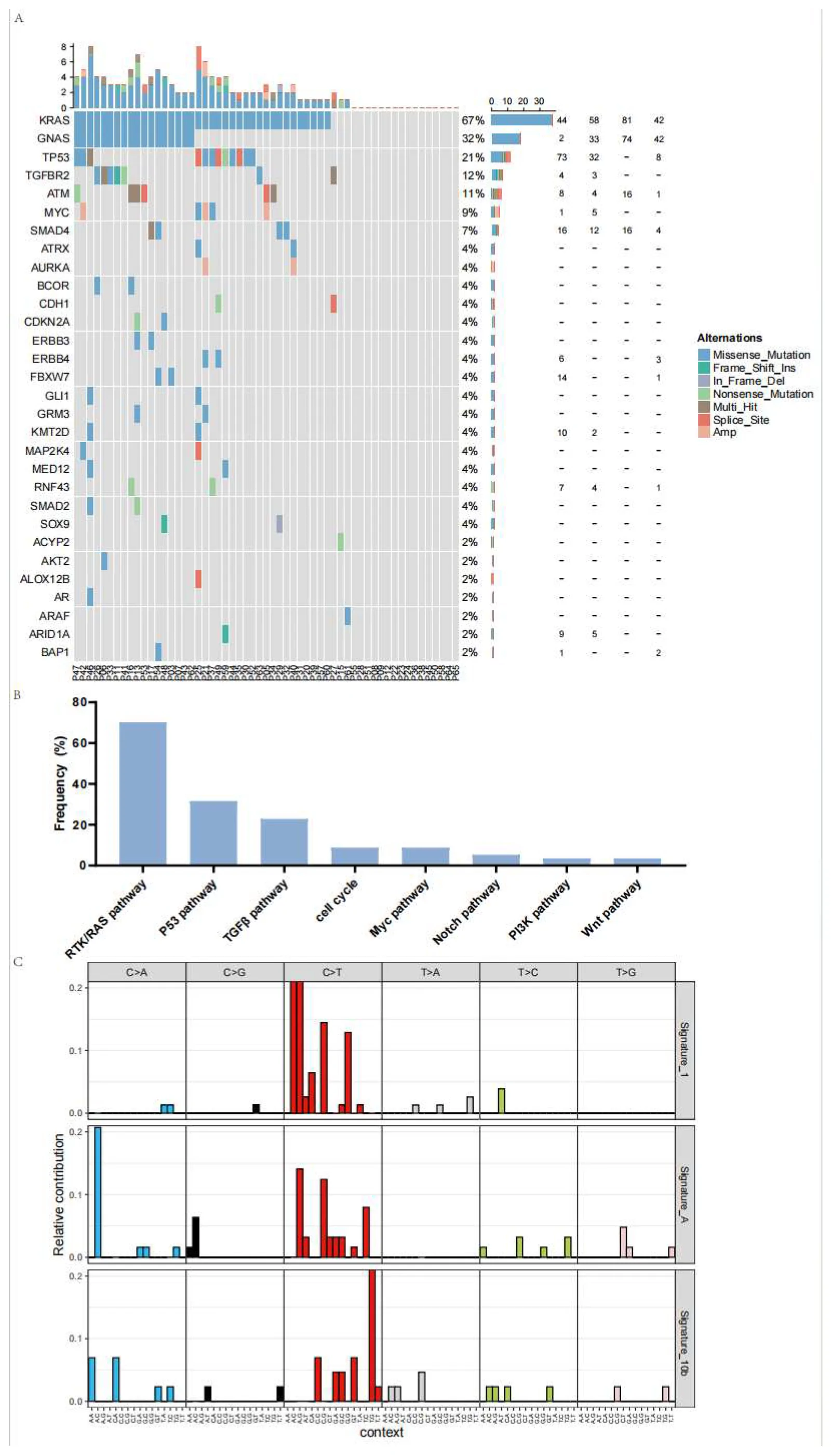
Genomic landscape of PMP. (**A**) Comparison of the mutation frequencies of recurrently mutated genes between Chinese patients with PMP and previously reported cohorts from other populations. (**B**) Distribution of alterations across canonical oncogenic signaling pathways. (**C**) Mutational signature profile of Chinese patients with PMP based on single-base substitution (SBS) patterns.

The genomic landscape of PMP was dominated by recurrent *KRAS* and *GNAS* alterations. In this Chinese cohort, 77.2% of patients harbored at least one somatic alteration, with *KRAS* (66.7%), *GNAS* (31.6%), and *TP53* (21.1%) representing the most frequently mutated genes (Figure 2A). To compare genomic characteristics across populations, we analyzed previously reported PMP cohorts [15,16,17]. *KRAS* and *GNAS* were consistently among the most frequently altered genes, although their mutation frequencies varied across cohorts, suggesting population-level genomic differences (Figure 2A). Compared with colorectal cancer (CRC), *GNAS* mutations were significantly enriched in PMP, whereas *KRAS* alterations were detected in both malignancies with different frequencies. CRC exhibited a distinct genomic profile characterized by frequent *APC* and *TP53* mutations, highlighting differences in the observed genomic landscapes. At the pathway level, alterations in the RTK/RAS pathway (70.2%) were most common, followed by the TP53 pathway (31.6%) and TGF-β pathway (22.8%) (Figure 2B). No alterations were observed in the Nrf2 or Hippo pathways. Mutational signature analysis identified Signature 1, associated with spontaneous deamination of 5-methylcytosine, and Signature 10, associated with polymerase epsilon exonuclease domain mutations, as the main signatures identified in this exploratory analysis. An additional profile, designated Signature A, showed a maximum cosine similarity below 0.80 to the COSMIC v3.4 SBS reference signatures and was classified as unmatched under the threshold used in this analysis (Figure 2C).

### 3.3. Concordance of Genomic Alterations Across Specimen Types

**Figure 3:**
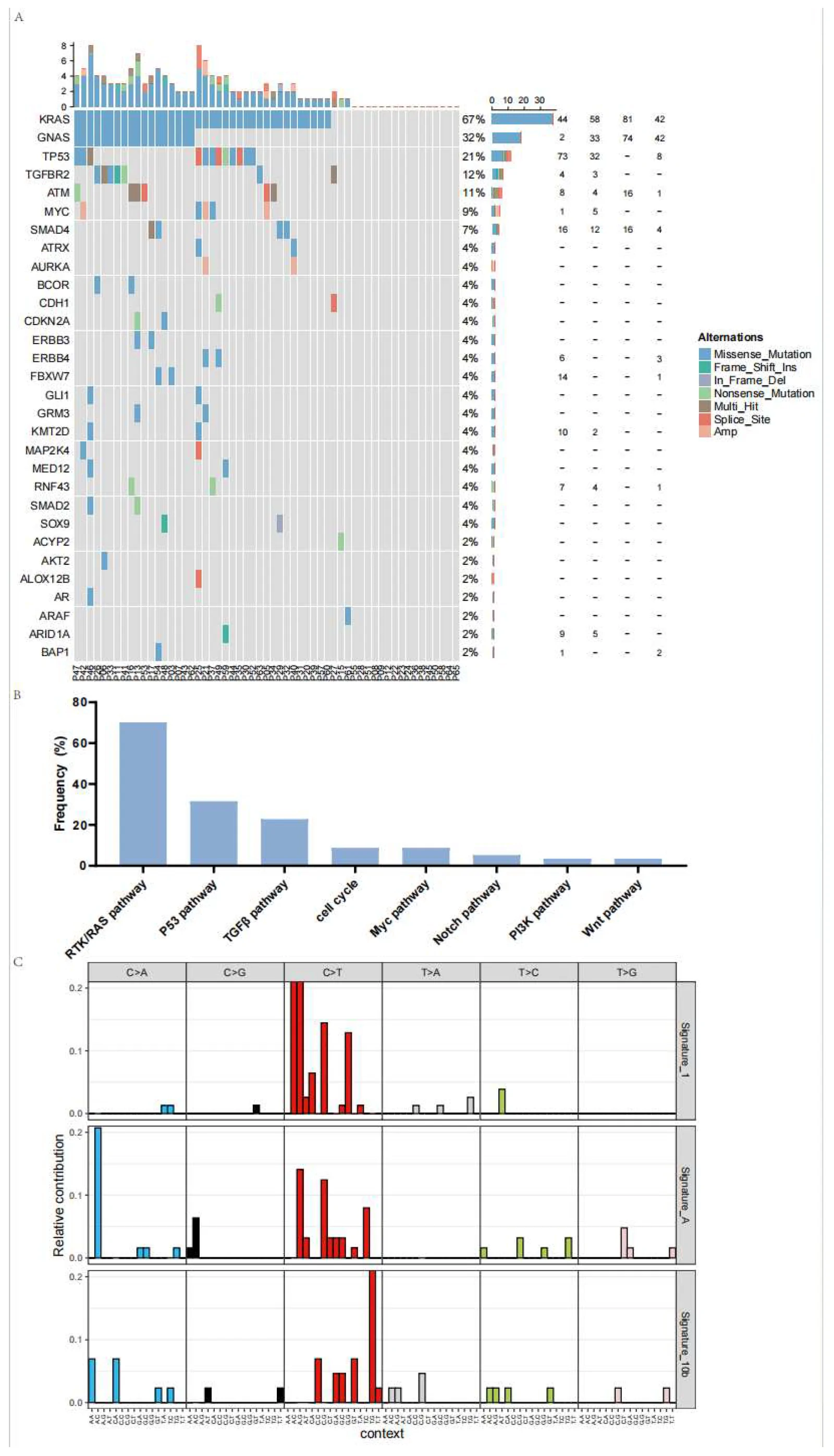
Concordance of genomic alterations among tissue, ascites cfDNA, and plasma ctDNA samples. (**A**) Heatmap showing the distribution of shared and sample-specific mutated genes detected in the three specimen types. (**B**) Bar plot showing the proportion of shared and sample-specific mutations for each patient. (**C**) UpSet plot illustrating the overlap of genomic alterations detected amongthe three specimen types. Each bar represents the number of mutations shared by the indicated sample combinations.

To evaluate genomic concordance among different biospecimens, 28 patients with matched tumor tissue, ascitic fluid, and plasma samples were analyzed using genomic regions shared across sequencing platforms. The overall mutational landscape was comparable among specimen types, with *KRAS*, *GNAS*, and *TP53* consistently representing the most frequently altered genes (Figure 3A; Figure S1). However, mutation detection varied substantially across specimen types. Somatic alterations were detected in 89.3% (25/28) of ascites samples, compared with 67.9% (19/28) of tissues samples and 42.9% (12/28) of plasma samples. Patient-level concordance across all three specimen types was 35.7% (10/28). Specifically, 28.6% (8/28) had at least one identical alteration detected in all three specimen types, whereas 7.1% (2/28) had no detectable alterations in any of the three specimens (Figure 3A,B).

At the mutation level, 95 unique alterations were identified across the three specimen types, of which 22.1% (21/95) were detected in all three types. Alterations shared between tissue and ascites accounted for 56.8% (54/95) of all unique alterations, whereas 22.1% (21/95) were shared between tissue and plasma and 24.2% (23/95) between ascites and plasma. Ascites contained the largest proportion of specimen-specific alterations, with 34.7% (33/95) detected exclusively in ascitic fluid, compared with 4.2% (4/95) in tumor tissue and 2.1% (2/95) in plasma (Figure 3C). Using tumor tissue as the reference, ascites showed higher mutation-level PPA than plasma (93.1% [95% CI, 83.6–97.3%] vs. 36.2% [95% CI, 25.1–49.1%], respectively).

### 3.4. Concordance Analysis Between Ascites and Tumor Tissue

**Figure 4:**
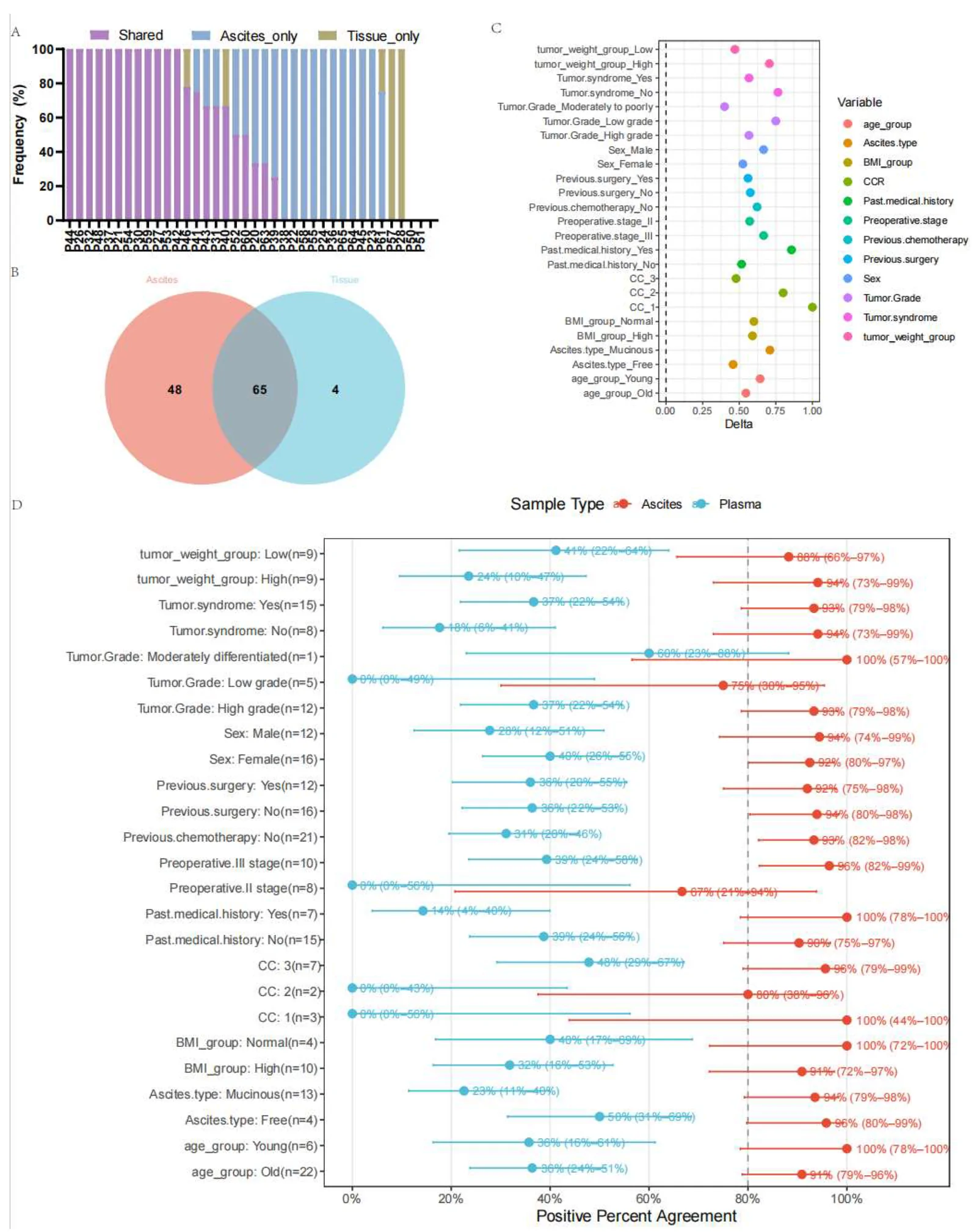
Concordance analysis of ctDNA detection. (**A**) Bar plot showing the proportions of shared and sample-specific mutations for each patient. (**B**) Venn diagram illustrating the overlap of genomic alterations identified among tissue and ascites cfDNA. (**C**) ΔPPA analysis showing the difference in PPA between ascites cfDNA and plasma ctDNA relative to matched tissue samples across clinical subgroups. Positive ΔPPA values indicate higher concordance for ascites than for plasma. (**D**) Forest plots showing the PPA between liquid biopsy samples (ascites cfDNA and plasma ctDNA) and matched tissue samples across clinical subgroups. Points represent the estimated PPA and horizontal bars indicate the 95% confidence intervals. The dashed vertical line denotes a PPA threshold of 80%, indicating high concordance.

To further assess genomic concordance between ascites and tumor tissue, we analyzed an expanded cohort of 37 patients with matched ascites–tissue samples. Among these patients, 59.5% showed identical detected mutation profiles between the two specimen types, whereas 8.1% had no detectable alterations in either specimen. Notably, 27.0% harbored alterations detected in ascites but not in the matched tissue sample (Figure 4A; Figure S2). Using tumor tissue as the reference, ascites showed a high overall PPA of 94.2% (Figure 4B), indicating that most tissue-detected alterations were also detected in ascites. Ascites profiling identified a greater number of genomic alterations than tissue profiling (113 vs. 71).

Gene-level agreement showed a similar pattern. PPA ranged from 95.5% to 100% across the 10 recurrently altered genes, indicating high recovery of tissue-detected alterations in ascites. However, NPA varied substantially across genes, most notably for *KRAS* (26.6%, 4/15) and *GNAS* (69.0%, 20/29), whereas NPA was higher for the remaining genes (83.3–100%). The low NPA for *KRAS* reflected frequent detection of *KRAS* alterations in ascites when the matched tissue sample was negative (11/15 tissue-negative cases). Accordingly, overall percentage agreement was also lower for *KRAS* (67.6%) and *GNAS* (75.7%) than for the other recurrently altered genes (Table 2). Variant allele frequencies were generally higher in ascites cfDNA than in tumor tissue (Figure S3).

### 3.5. Subgroup Concordance Analysis

To evaluate whether clinical characteristics affected genomic concordance, subgroup analyses were performed across different clinicopathological categories. Tumor tissue was used as the reference specimen for calculating positive percent agreement (PPA), and ascites cfDNA and plasma ctDNA were evaluated separately against tissue-based mutation detection. Therefore, tumor tissue was not included as an independent comparison group in the subgroup analyses. Ascites cfDNA maintained high concordance with tumor tissue across most subgroups, with consistently high PPA values (Figure 4D). In contrast, plasma ctDNA showed lower and more variable concordance across subgroups (Figure 4C,D). Lower PPA values were observed in patients with low-grade tumors and stage II disease. In these subgroups, plasma ctDNA showed no detectable concordant mutations (PPA = 0% for both), whereas ascites cfDNA retained partial concordance (PPA = 75% and 67%, respectively). CC status showed a potential association with concordance, suggesting that tumor burden may affect ctDNA release and detection. However, patient-level concordance rates did not significantly differ among clinical subgroups (Supplementary Table S3).

### 3.6. Molecular Characteristics Across Kras Mutation Subgroups

**Figure 5:**
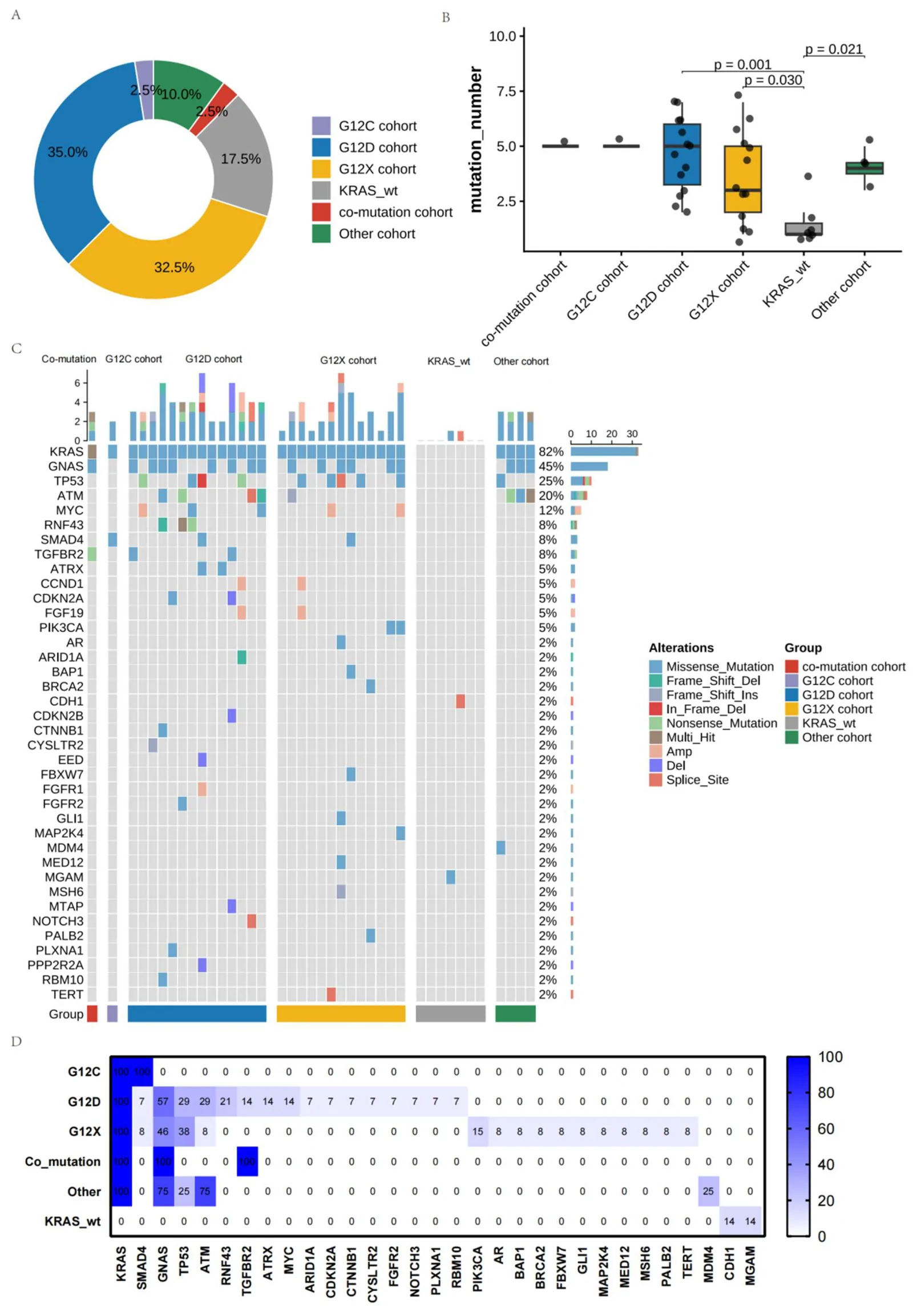
Molecular characteristics of KRAS-defined subgroups based on ascites genomic profiling. (**A**) Distribution of KRAS subgroups in the ascites cohort. The proportions of patients harboring different KRAS mutation subgroups, including G12C, G12D, G12X, co-mutation, other mutations, and KRAS-wt, are shown. (**B**) Comparison of the numbers of detected genomic alterations across KRAS-defined subgroups. Each point represents the total alteration count in an individual sample. (**C**) Oncoplot depicting the mutational landscape across KRAS molecular subgroups. Each column represents an individual patient and each row represents a gene. Colored tiles indicate distinct types of genomic alterations. The bar plot above the heatmap indicates the total mutation count per sample, while the bar plot on the right shows the overall mutation frequency of each gene in the cohort. (**D**) Heatmap summarizing gene mutation frequencies across KRAS subgroups. The color intensity represents the proportion of samples harboring mutations in each gene within the corresponding subgroup, with darker colors indicating higher mutation frequencies.

To characterize molecular differences among KRAS subgroups, 40 patients with available ascites genomic profiling were analyzed. KRAS alterations were classified into G12D (35.0%), G12X (32.5%), KRAS wild-type (KRAS-wt, 17.5%), and other subgroups, whereas G12C mutations were uncommon (2.5%). One patient with a G12D mutation accompanied by additional alterations was classified as the co-mutation subgroup (Figure 5A). Given the small sample sizes of several subgroups, particularly the G12C and co-mutation subgroups, which each included only one patient, comparisons involving these subgroups were not considered informative for statistical inference. Accordingly, the subgroup findings presented below should be interpreted as exploratory, with genomic differences primarily described rather than used to establish subgroup-specific characteristics. 

KRAS-wt tumors exhibited lower mutation burden than KRAS-mutant tumors, including the G12D (*p* = 0.001), G12X (*p* = 0.030), and other mutation subgroups (*p* = 0.021, Figure 5B). Mutational profiles differed substantially across KRAS subgroups. No shared mutated genes were identified between KRAS-wt and KRAS-mutant tumors (Figure 5C,D). Several alterations showed subgroup-specific distributions: *ATRX*, *MYC*, and *ARID1A* mutations were detected exclusively in the G12D subgroup, whereas *PIK3CA*, *BRCA2*, *BAP1*, and *MED12* alterations were restricted to the G12X subgroup. MDM4 mutations were observed only in the other subgroup, while *CDH1* and *MGAM* mutations were limited to KRAS-wt tumors.

### 3.7. Landscape of Oncogenic Pathways and Clinically Actionable Alterations Across Kras Molecular Subgroups

**Figure 6:**
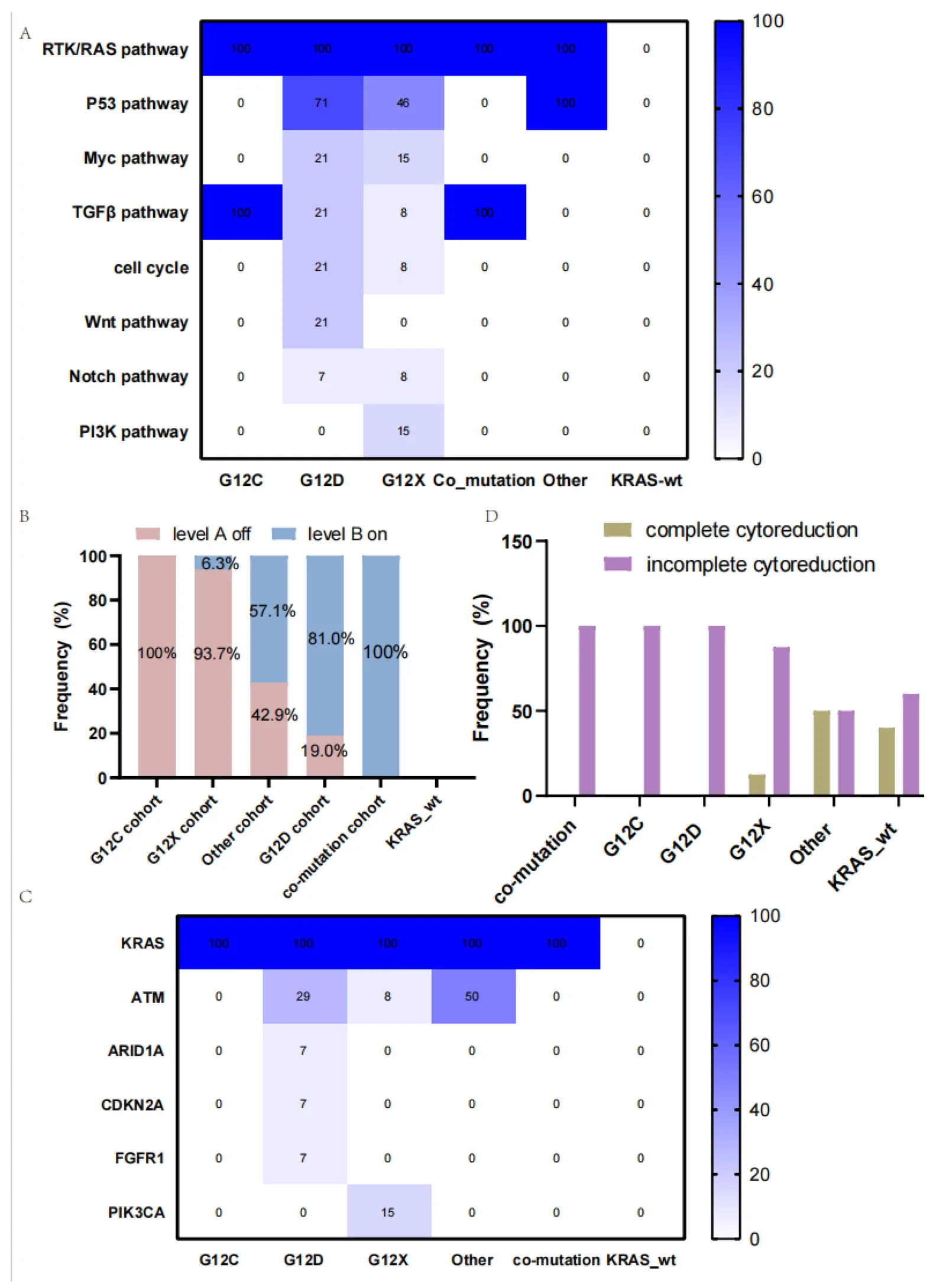
Oncogenic pathway alterations and distribution of clinically actionable alterations across KRAS molecular subgroups. (**A**) Distribution of oncogenic pathway alterations among KRAS-defined subgroups. (**B**) Distribution of clinically actionable alterations across KRAS molecular subgroups based on evidence levels defined by the OncoKB database. (**C**) Frequencies of clinically actionable genes across KRAS molecular subgroups, illustrating subgroup-specific distributions of actionable genomic alterations. (**D**) Distribution of cytoreduction outcomes across molecularly defined KRAS subgroups. The *x*-axis represents the KRAS subgroups, and the *y*-axis indicates the proportion of patients (%). Complete cytoreduction (CC0) is indicated in purple, whereas incomplete cytoreduction (CC1–CC3) is indicated in yellow.

We performed an exploratory analysis of alterations in canonical oncogenic pathways and potentially actionable events across KRAS-defined subgroups. Given the small subgroup sizes, including subgroups with a single patient, the findings were summarized descriptively. KRAS-mutant tumors displayed heterogeneous pathway alteration patterns, whereas no canonical pathway alterations were detected in the KRAS-wt subgroup. The G12D and G12X subgroups shared alterations involving the TP53, MYC, TGF-β, cell cycle, and Notch pathways, but also showed distinct pathway preferences. Specifically, Wnt pathway alterations were exclusively observed in the G12D subgroup, whereas PI3K pathway alterations were restricted to the G12X subgroup. Alterations in the other KRAS-mutant subgroup were mainly limited to the RTK/RAS and TP53 pathways (Figure 6A).

Clinically actionable alterations, classified according to OncoKB evidence levels, varied across KRAS subgroups (Figure 6B). Level A (OncoKB Levels 1–2) represented FDA-approved or guideline-supported biomarkers, whereas Level B (Levels 3–4) indicated potential actionability supported by clinical or biological evidence. On-label and off-label therapies referred to drugs approved for PMP and other cancer types, respectively. Level A alterations were predominant in the G12C and G12X subgroups (100% and 93.7%, respectively), whereas Level B alterations accounted for most actionable events in the G12D and co-mutation subgroups (81.0% and 100%, respectively). No alterations classified as potentially actionable under the applied annotation criteria were identified in KRAS-wt tumors. At the gene level, potentially actionable alterations were mainly observed in *KRAS*, *ATM*, *CDKN2A*, *FGFR1*, *ARID1A*, and *PIK3CA* (Figure 6C). The frequency of potential alterations was higher in the G12D and G12X subgroups. In the G12D subgroup, *ATM* was the most frequently altered actionable gene (28.6%), followed by *ARID1A*, *CDKN2A*, and *FGFR1* (each 7.1%). In the G12X subgroup, actionable alterations involved *PIK3CA* (15.4%) and *ATM* (7.7%), with *PIK3CA* alterations detected exclusively in this subgroup.

Clinical outcomes also differed according to KRAS status. The distribution of CC status across KRAS-defined subgroups is shown in Figure 6D. Complete cytoreduction was defined as CC0, whereas CC1–CC3 were classified as incomplete cytoreduction. Given the small and uneven subgroup sizes, these findings were interpreted descriptively rather than as evidence of KRAS-associated differences in surgical outcomes. Further examination of KRAS-wt patients revealed no detectable mutations among those classified as having a complete cytoreduction. In contrast, three patients with an incomplete cytoreduction harbored distinct alterations in *ATM* (p.Y583C), *CDH1* (splice-site mutation), and MGAM (p.R1098L). These observations suggest genomic heterogeneity within the KRAS-wt subgroup but remain exploratory given the limited sample size.

### 3.8. Gene Co-Occurrence and Mutual Exclusivity Across Kras Subgroups

Co-occurrence and mutual exclusivity analyses revealed distinct mutation patterns across KRAS subgroups. A significant co-occurrence between GNAS and TP53 mutations was observed in the G12D subgroup (Figure S4A). No significant co-occurrence or mutual exclusivity events were identified in the G12X or other subgroups (Figure S4B,C).

### 3.9. Clinical Correlates of Kras Molecular Subgroups

Clinicopathological characteristics across KRAS-defined subgroups are summarized in Table 3. Mucinous ascites was more frequently observed in the G12D (76.9%) and G12X (66.7%) subgroups, whereas KRAS-wt tumors more frequently presented with free ascites or absence of ascites. Among patients with available tumor-marker measurements, preoperative CA125 and CA19-9 levels were descriptively higher in the G12D subgroup and lower in the KRAS-wt subgroup. Similar descriptive patterns were observed for postoperative CA19-9 and ascitic tumor markers, including CA125, CEA, and CA19-9. CK7 expression also varied descriptively across the subgroups, with no CK7-positive cases identified among evaluable KRAS-wt tumors. None of the examined clinicopathological characteristics differed significantly among the three major KRAS subgroups. Given the small subgroup sizes and missing observations for several clinical variables, these findings were considered exploratory and should not be interpreted as evidence of KRAS subtype-specific clinical characteristics.

## 4. Discussion

PMP presents substantial challenges for liquid-biopsy-based molecular profiling because of its distinctive biological and clinicopathological characteristics. In this study, multispecimen genomic profiling revealed considerable heterogeneity across tumor tissue, ascites, and plasma. Patient-level concordance across all three specimen types was 35.7%, comprising patients with at least one identical alteration detected across all three specimen types (28.6%) and those with no detectable alterations in any specimen (7.1%). Plasma showed limited recovery of tissue-detected alterations, whereas ascites demonstrated substantially higher tissue-referenced PPA and identified additional alterations not detected in matched tissue specimens. These findings suggest that ascites may provide a complementary source of molecular information in PMP. Exploratory analyses further revealed distinct genomic and pathway-level characteristics across KRAS-defined subgroups, underscoring the molecular heterogeneity of this disease.

Tumor tissue is a conventional source for genomic profiling, but its use may be constrained by invasive sampling and incomplete representation of spatial heterogeneity [18]. These limitations are particularly relevant in PMP, where abundant extracellular mucin and low neoplastic cellularity may compromise tissue-based molecular profiling [6,7]. Although plasma ctDNA has transformed molecular profiling in many solid tumors [19,20,21,22], utility appears to be more limited in PMP [8], with low plasma–tissue concordance reported in peritoneal malignancies [23] and low ctDNA detection rates in metastatic appendiceal adenocarcinoma [24]. Collectively, these findings suggest that tumor-derived alterations may be less readily detectable in plasma in PMP and other predominantly peritoneal malignancies, although the biological and technical determinants of this limited detectability remain incompletely understood. By contrast, ascitic fluid represents a tumor-proximal compartment that may contain molecular material derived from multiple peritoneal lesions [25]. In our study, ascites recovered a substantially greater proportion of tissue-detected alterations than plasma while also containing the largest proportion of specimen-specific alterations. This combination of high tissue-referenced PPA and additional ascites-detected alterations suggests that ascitic fluid may provide molecular information complementary to that obtained from a localized tissue specimen. Moreover, tissue-based profiling is inherently restricted to the sampled lesion and may therefore fail to capture genomic alterations arising from spatially heterogeneous peritoneal deposits [26]. Nevertheless, alterations detected exclusively in ascites should be interpreted cautiously. Ascites and plasma samples were sequenced at a depth of >10,000× with a VAF threshold of ≥0.1%, whereas tissue samples were sequenced at >1000× with a VAF threshold of ≥1%. The higher sequencing depth and lower VAF threshold used for liquid biopsy specimens may have contributed to the greater number of alterations detected in ascites and the observed inter-specimen discordance. Ascites-only alterations could therefore reflect spatial tumor heterogeneity, tissue sampling limitations, or differences in analytical sensitivity. Without systematic orthogonal validation or additional tissue sampling, their origin remains uncertain. Thus, our findings support further evaluation of ascites as a complementary biospecimen for molecular profiling in PMP.

Exploratory KRAS stratification revealed variation in the distribution of genomic alterations within PMP. Wnt pathway alterations were detected in the G12D subgroup, whereas PI3K pathway alterations were observed in the G12X subgroup. However, the small and highly imbalanced subgroup sizes preclude firm conclusions about subgroup-specific pathway enrichment or biological differences. These patterns may reflect chance variation, differences in case composition, or technical factors and should be regarded as hypothesis-generating. Previous studies have linked KRAS G12D-associated signaling to Wnt pathway activity and have reported context-dependent differences in sensitivity to cytotoxic, targeted, and immune-based therapies [27,28,29]. However, these findings cannot be directly extrapolated to PMP, and the present subgroup analyses are insufficient to establish similar biological or therapeutic associations. In our cohort, KRAS-wt tumors harbored fewer detected genomic alterations and exhibited a distinct alteration profile compared with KRAS-mutant tumors, suggesting potential differences in their underlying molecular biology. Alterations not captured by the targeted sequencing panel, including structural variants, transcriptional changes, and epigenetic dysregulation, may contribute to the biology of KRAS-wt PMP. Broader genomic and multi-omics analyses will therefore be needed to characterize the molecular mechanisms underlying these tumors.

The distribution of potentially clinically actionable alterations also varied across KRAS-defined subgroups. Alterations involving KRAS, ATM, CDKN2A, FGFR1, ARID1A, and PIK3CA were predominantly identified in KRAS-mutant tumors. These findings suggest heterogeneity in the landscape of potentially targetable alterations across molecular subgroups. Although several of these genes have been linked to approved or investigational targeted strategies in other tumor types [30,31,32,33], their therapeutic relevance in PMP remains to be established. Notably, the absence of actionable alterations identified by the present panel in KRAS-wt tumors does not exclude other therapeutically relevant molecular abnormalities that may not be captured by targeted sequencing. However, these findings cannot be directly extrapolated to PMP, and the present subgroup analyses are insufficient to establish similar biological or therapeutic associations.

Several limitations should be acknowledged. First, this retrospective, single-center study included a relatively small cohort, with uneven KRAS subgroup sizes and some subgroups comprising only one patient, limiting the reliability and generalizability of subgroup comparisons. The cohort was also heterogeneous in histopathological grade, disease status, and prior treatment, which may have influenced molecular and clinical comparisons. Second, specimen availability varied among patients, resulting in smaller matched subsets and potential selection bias associated with the requirement for available NGS data. Third, missing clinical data and incomplete follow-up limited subgroup comparisons and precluded robust survival analyses. Fourth, targeted sequencing may not capture the full molecular complexity of PMP, including structural variants, transcriptomic alterations, and epigenetic changes. Furthermore, mutational signature analysis was based on 235 SNVs obtained from targeted sequencing. The limited mutation counts and genomic coverage may affect the stability of signature extraction and the reliability of signature assignment. These results should therefore be interpreted as exploratory rather than definitive evidence of the underlying mutational processes in PMP. Fifth, differences in sequencing depth, VAF thresholds, and targeted panel coverage between tissue and liquid biopsy samples may have influenced variant detection and inter-specimen comparisons. Consequently, specimen-specific alterations cannot be attributed solely to biological heterogeneity. Finally, additional alterations detected in ascites were not systematically validated using orthogonal methods; therefore, whether these discordant alterations reflect spatial heterogeneity, differences in tumor-derived DNA abundance, or analytical factors remains uncertain. Despite these limitations, our study provides a multispecimen characterization of genomic heterogeneity in PMP and demonstrates marked differences in alteration detection across tumor tissue, ascites, and plasma. The high recovery of tissue-detected alterations in ascites, together with additional alterations not detected in matched tissue, supports further investigation of ascitic fluid as a complementary biospecimen for molecular profiling. The exploratory KRAS subgroup findings provide hypotheses for future investigation, but their biological and clinical relevance remains to be established.

## 5. Conclusions

Multispecimen genomic profiling revealed substantial inter-specimen heterogeneity in pseudomyxoma peritonei. Ascites captured most alterations detected in matched tumor tissue and revealed additional alterations absent from tissue, supporting its potential use as a complementary biospecimen, particularly when tissue availability is limited. The exploratory KRAS subgroup findings provide hypotheses for future investigation, but their biological and clinical relevance remains to be established. Prospective studies with standardized sampling, orthogonal validation, and clinical outcome data are needed to confirm these findings and establish their potential diagnostic and therapeutic relevance.

## Figures and Tables

**Table 1:** Baseline clinical characteristics of patients.

Characteristic	*n* = 60
Demographic characteristics	
Age, years, median [range]	57 (50–79)
Sex (Male: Female)	21:39 (35% vs. 65%)
BMI, kg/m^2^, median [range] (*n* missing = 17)	23.4 (22.3–26.4)
Primary tumor origin (*n* missing = 13)	
Appendiceal	41 (87.2)
Ovarian	2 (4.3%)
Gastrointestinal, unspecified	2 (4.3%)
Colonic	1 (2.1%)
Urachal	1 (2.1%)
Medical history (*n* missing = 8)	
Hypertension	9 (17.6%)
Diabetes mellitus	2 (3.9%)
Both hypertension and diabetes mellitus	1 (2.0%)
Hepatitis	1 (2.0%)
No	38 (74.5%)
Preoperative status	
ECOG performance status ≥ 2 (*n* missing = 9)	28 (54.0%)
Stage III disease (*n* missing = 17)	28 (65.1%)
Previous treatment	
Prior surgery at another hospital	32 (53.3%)
Prior chemotherapy	7 (11.7%)
Prior HIPEC	6 (10.0%)
No previous treatment	27 (45.0%)
Treatment (*n* missing = 10)	
CRS + HIPEC	43 (86%)
CRS alone	7 (14%)
CC-0/1 resection (*n* missing = 17)	12 (27.9%)
Cytoreduction outcomes (*n* missing = 17)	
Incomplete cytoreduction	37 (86.0%)
Complete response	6 (14.0%)
Tumor markers, median (IQR) (*n* missing = 28)	
CA-125	51 (19–119)
CEA	6 (4–30)
CA19-9	79 (15–400)
High-grade peritoneal pseudomyxoma (*n* missing = 12)	31 (64.6%)
Ascites characteristics (*n* missing = 3)	
Mucinous ascites	36 (63.1%)
Free ascites	12 (21.1%)
No ascites	9 (15.8%)
Tumor cell content (*n* missing = 3)	
<5%	7 (12.3%)
5–20%	37 (64.9%)
>20%	13 (22.8%)

**Table 2:** Top 10 genes: ascites and tissue consistency.

Patients Who Had Both Ascites and Tissue NGS (*n* = 37)						
		Ascites	Ascites	PPA	NPA	OPA
		+	-			
*KRAS*	Tissue +	21	1	95.5% (21/22)	26.6% (4/15)	67.6% (25/37)
	Tissue -	11	4			
*GNAS*	Tissue +	8	0	100% (8/8)	69.0% (20/29)	75.7% (28/37)
	Tissue -	9	20			
*TP53*	Tissue +	8	0	100% (8/8)	93.1% (27/29)	94.6% (35/37)
	Tissue -	2	27			
*ATM*	Tissue +	1	0	100% (1/1)	83.3% (30/36)	83.8% (31/37)
	Tissue -	6	30			
*MYC*	Tissue +	3	0	100% (3/3)	94.1% (32/34)	94.6% (35/37)
	Tissue -	2	32			
*TGFBR2*	Tissue +	3	0	100% (3/3)	100% (34/34)	100% (37/37)
	Tissue -	0	34			
*SMAD4*	Tissue +	2	0	100% (2/2)	97.1% (34/35)	97.3% (36/37)
	Tissue -	1	34			
*RNF43*	Tissue +	1	0	100% (1/1)	94.4% (34/36)	94.6% (35/37)
	Tissue -	2	34			
*ATRX*	Tissue +	1	0	100 (1/1)	97.2% (35/36)	97.3% (36/37)
	Tissue -	1	35			
*PIK3CA*	Tissue +	1	0	100 (1/1)	97.2% (35/36)	97.3% (36/37)
	Tissue -	1	35			

+, positive; -, negative.

**Table 3:** Clinicopathological characteristics across among different KRAS groups.

Characteristics	Co-Mutation Cohort	G12C Cohort	G12D Cohort	G12X Cohort	Other Cohort	KRAS_wt	*p*-Value
	N = 1	N = 1	N = 14	N = 13	N = 4	N = 7	
Sex							0.778
Male	0 (0.0%)	1 (100.0%)	5 (35.7%)	6 (46.2%)	1 (25.0%)	2 (38.6%)	
Female	1 (100.0%)	0 (0.0%)	9 (64.3%)	7 (53.8%)	3 (75.0%)	5 (71.4%)	
Age	40 (-)	53 (-)	56 (14.3)	59 (13.0)	53 (12.8)	59 (8.4)	0.771
Tumor syndrome (missing = 6)							0.769
Absent	0 (0.0%)	0 (0.0%)	4 (36.4%)	4 (36.4%)	0 (0.0%)	1 (16.7%)	
Present	1 (100.0%)	1 (100.0%)	7 (63.6%)	7 (63.6%)	4 (100.0%)	5 (83.3%)	
Past medical history (missing = 8)							0.439
Hepatitis	0 (0.0%)	0 (0.0%)	1 (9.1%)	0 (0.0%)	0 (0.0%)	0 (0.0%)	
Hypertension	0 (0.0%)	0 (0.0%)	2 (18.2%)	3 (27.3%)	1 (25.0%)	0 (0.0%)	
Diabetes mellitus	1 (100.0%)	0 (0.0%)	0 (0.0%)	0 (0.0%)	0 (0.0%)	0 (0.0%)	
None	0 (0.0%)	1 (100.0%)	8 (72.7%)	7 (63.6%)	3 (75.0%)	5 (100.0%)	
Previous chemotherapy (missing = 7)							0.778
No	1 (100.0%)	1 (100.0%)	9 (81.8%)	10 (90.9)	3 (75.0%)	5 (100.0%)	
Yes	0 (0.0%)	0 (0.0%)	2 (18.2%)	1 (9.1%)	1 (25.0%)	0 (0.0%)	
Previous intraperitoneal perfusion (missing = 7)							0.354
No	1 (100.0%)	1 (100.0%)	10 (90.9%)	11 (100.0%)	3 (75.0%)	5 (100.0%)	
Yes	0 (0.0%)	0 (0.0%)	1 (9.1%)	0 (0.0%)	1 (25.0%)	0 (0.0%)	
Previous surgery (missing = 1)							>0.999
No	0 (0.0%)	1 (100.0%)	7 (50.0%)	7 (53.8%)	2 (50.0%)	3 (50.0%)	
Yes	1 (100.0%)	0 (0.0%)	7 (50.0%)	6 (46.2%)	2 (50.0%)	3 (50.0%)	
Preoperative stage (missing = 16)							0.358
Stage I	0 (0.0%)	0 (0.0%)	0 (0.0%)	0 (0.0%)	1 (33.3%)	2 (50.0%)	
Stage II	0 (0.0%)	0 (0.0%)	3 (42.9%)	2 (25.0%)	0 (0.0%)	1 (25.0%)	
Stage III	1 (100.0%)	1 (100.0%)	4 (57.1%)	6 (75.0%)	2 (66.7%)	1 (25.0%)	
Tumor weight (missing = 13)	2 (-)	2 (-)	3 (2.0)	2 (0.9)	2 (0.8)	1 (0.1)	0.386
Ascites type (missing = 3)							0.049
None	0 (0.0%)	0 (0.0%)	0 (0.0%)	2 (16.7%)	0 (0.0%)	2 (33.3%)	
Free ascites	0 (0.0%)	1 (100.0%)	3 (23.1%)	2 (16.7%)	3 (75.0%)	3 (50.0%)	
Mucinous ascites	1 (100.0%)	0 (0.0%)	10 (76.9%)	8 (66.7%)	1 (25.0%)	1 (16.7%)	
Postoperative CCR score (missing = 16)							0.857
CCR-0	0 (0.0%)	0 (0.0%)	0 (0.0%)	1 (12.5%)	1 (33.3%)	2 (40.0%)	
CCR-1	0 (0.0%)	0 (0.0%)	2 (28.6%)	1 (12.5%)	1 (33.3%)	2 (40.0%)	
CCR-2	0 (0.0%)	0 (0.0%)	1 (14.3%)	2 (25.0%)	0 (0.0%)	0 (0.0%)	
CCR-3	1 (100.0%)	1 (100.0%)	4 (57.1%)	4 (50.0%)	1 (33.3%)	1 (20.0%)	
Intraoperative HIPEC (missing = 5)							0.879
No	0 (0.0%)	0 (0.0%)	2 (18.2%)	1 (8.3%)	1 (25.0%)	1 (16.7%)	
Yes	1 (100.0%)	1 (100.0%)	9 (81.8%)	11 (91.7%)	3 (75.0%)	5 (83.3%)	
Preoperative CA_125 (missing = 21)	55 (-)	101 (-)	180 (123.9)	66 (49.1)	43 (49.3)	33 (36.7)	0.297
Missing	0	0	9	5	2	5	
Preoperative _CEA (missing = 21)	14 (-)	6 (-)	29 (29.8)	6 (4.2)	31 (41.0)	2 (0.8)	0.17
Preoperative CA_199 (missing = 21)	369 (-)	34 (-)	3068 (5985.3)	203 (272.9)	194 (264.9)	24 (18.4)	0.88
Postoperative CA_125 (missing = 17)	56 (-)	187 (-)	87 (75.1)	84 (38.5)	34 (18.0)	29 (16.4)	0.113
Postoperative_CEA (missing = 17)	8 (-)	4 (-)	15 (14.2)	20 (46.0)	8 (10.4)	4 (4.6)	0.721
Postoperative CA_199 (missing = 17)	211 (-)	27 (-)	723 (1263.2)	218 (336.3)	72 (111.2)	6 (6.1)	0.207
Ascites CA_125 (missing = 11)	440 (-)	394 (-)	286 (280.1)	365 (329.6)	603 (585.5)	283 (333.3)	0.861
Ascites_CEA (missing = 11)	1382 (-)	2909 (-)	5342 (8108.3)	5451 (10,901.6)	2830 (4132.1)	487 (733.1)	0.56
Ascites CA_199 (missing = 11)	11,808 (-)	533 (-)	3259 (6538.0)	4474 (7691.8)	2417 (3830.3)	657 (1453.9)	0.309
CK7 expression (missing = 20)							0.76
Negative	0 (0.0%)	1 (100.0%)	4 (66.7%)	4 (50.0%)	1 (33.3%)	1 (100.0%)	
Positive	1 (100.0%)	0 (0.0%)	2 (33.3%)	4 (50.0%)	2 (66.7%)	0 (0.0%)	
CK20 expression (missing = 17)							>0.999
Negative	0 (0.0%)	0 (0.0%)	0 (0.0%)	1 (10.0%)	0 (0.0%)	0 (0.0%)	
Positive	1 (100.0%)	1 (100.0%)	7 (100.0%)	9 (90.0%)	3 (100.0%)	1 (100.0%)	
CDX2 expression (missing = 16)							>0.999
Negative	0 (0.0%)	0 (0.0%)	0 (0.0%)	1 (10.0%)	0 (0.0%)	0 (0.0%)	
Positive	1 (100.0%)	1 (100.0%)	7 (100.0%)	9 (90.0%)	3 (100.0%)	2 (100.0%)	
MUC2 expression (missing = 16)							>0.999
Negative	0 (0.0%)	0 (0.0%)	0 (0.0%)	1 (10.0%)	0 (0.0%)	0 (0.0%)	
Positive	1 (100.0%)	1 (100.0%)	7 (100.0%)	9 (90.0%)	3 (100.0%)	2 (100.0%)	
HER2 expression (missing = 23)							>0.999
Negative	0 (-)	0 (0.0%)	1 (20.0%)	2 (28.6%)	0 (0.0%)	0 (0.0%)	
Positive	0 (-)	1 (100.0%)	4 (80.0%)	5 (71.4%)	2 (100.0%)	2 (100.0%)	
Ki67 expression (missing = 15)	-(-)	10 (-)	22 (24.9)	24 (18.7)	37 (25.2)	20 (13.2)	0.732
BMI (missing = 16)	26 (-)	24 (-)	23 (3.9)	25 (3.7)	27 (1.0)	22 (4.2)	0.475

“-” as “data not available”.

## Data Availability

The datasets used and/or analyzed during the current study are available from the corresponding author on reasonable request, subject to institutional and ethical restrictions related to patient privacy.

## Supplementary Materials

The following supporting information can be downloaded at: https://www.mdpi.com/article/10.3390/diagnostics16213463/s1, Figure S1. Comparison of mutation landscapes across different sample types. Heatmap illustrating the mutational profiles detected in ascites, tumor tissue, and plasma samples. Each column represents an individual sample, and each row represents a gene. Colored tiles indicate the presence of somatic mutations, enabling direct comparison of genomic alteration patterns among the three sample types. Figure S2. Comparison of mutation landscapes across different sample types. Heatmap illustrating the mutational profiles detected in ascites and tumor tissue samples. Each column represents an individual sample, and each row represents a gene. Colored tiles indicate the presence of somatic mutations, enabling direct comparison of genomic alteration patterns among the three sample types. Figure S3. Comparison of variant allele frequency (VAF) between tumor tissue and ascites samples. Figure S4. Gene co-occurrence and mutual exclusivity across KRAS mutation subgroups. (A–C) Analysis of gene co-occurrence and mutual exclusivity among recurrently mutated genes within different KRAS-defined cohorts, including the G12D cohort (A), G12X cohort (B), and other mutation cohort (C). Each panel illustrates pairwise relationships between mutated genes. Blue represents significant co-occurrence, yellow represents mutual exclusivity, and the size and color intensity indicate the strength of the association. Table S1. Genes shared between the 1299-gene and 272-gene sequencing panels. Table S2. Baseline clinical characteristics of patients. Table S3. The influence of different clinical factors on consistency.

## Abbreviations

PMP	Pseudomyxoma peritonei
HIPEC	Hyperthermic intraperitoneal chemotherapy
gDNA	Genomic DNA
SNV	Single-nucleotide variant
PPA	Positive percentage agreement
OPA	Overall percentage agreement
CRS	Cytoreductive surgery
NGS	Next-generation sequencing
BWA	Burrows–Wheeler Aligner
CNVs	Copy-number variants
NPA	Negative percentage agreement
CRC	Colorectal cancer
