# Supplementary material for: Multispecimen Genomic Profiling Reveals Inter-Specimen Heterogeneity and Supports Ascites as a Complementary Biospecimen in Pseudomyxoma Peritonei
Source: Diagnostics (Basel). 2026 Oct 23;16(21):3463. doi: 10.3390/diagnostics16213463 (PMC13761630; doi:10.3390/diagnostics16213463)
Supplement: Supplementary file 1 [file diagnostics-16-03463-s001.zip › Figure S1.pdf]

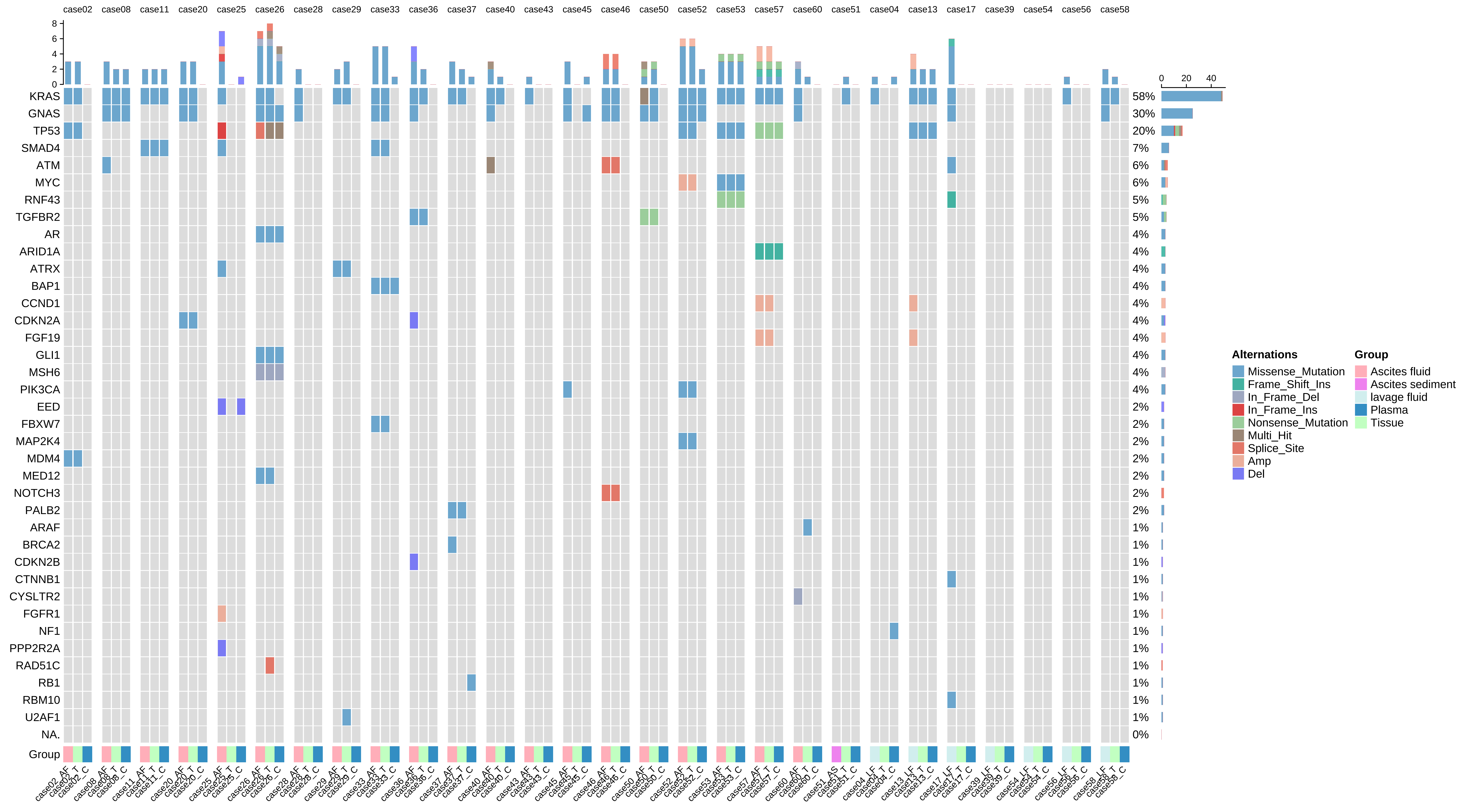

case02 case08 case11 case20 case25 case26 case28 case29 case33 case36 case37 case40 case43 case45 case46 case50 case52 case53 case57 case60 case51 case04 case13 case17 case39 case54 case56 case58
KRAS 58%
GNAS 30%
TP53 20%
SMAD4 7%
ATM 6%
MYC 6%
RNF43 5%
TGFBR2 5%
AR 4%
ARID1A 4%
ATRX 4%
BAP1 4%
CCND1 4%
CDKN2A 4%
FGF19 4%
GLI1 4%
MSH6 4%
PIK3CA 4%
EED 2%
FBXW7 2%
MAP2K4 2%
MDM4 2%
MED12 2%
NOTCH3 2%
PALB2 2%
ARAF 1%
BRCA2 1%
CDKN2B 1%
CTNNB1 1%
CYSLTR2 1%
FGFR1 1%
NF1 1%
PPP2R2A 1%
RAD51C 1%
RB1 1%
RBM10 1%
U2AF1 1%
NA. 0%
Group
Alternations
Missense_Mutation
Frame_Shift_Ins
In_Frame_Del
In_Frame_Ins
Nonsense_Mutation
Multi_Hit
Splice_Site
Amp
Del
Group
Ascites fluid
Ascites sediment
lavage fluid
Plasma
Tissue
