# Supplementary material for: Multispecimen Genomic Profiling Reveals Inter-Specimen Heterogeneity and Supports Ascites as a Complementary Biospecimen in Pseudomyxoma Peritonei
Source: Diagnostics (Basel). 2026 Oct 23;16(21):3463. doi: 10.3390/diagnostics16213463 (PMC13761630; doi:10.3390/diagnostics16213463)
Supplement: Supplementary file 1 [file diagnostics-16-03463-s001.zip › Figure S2.pdf]

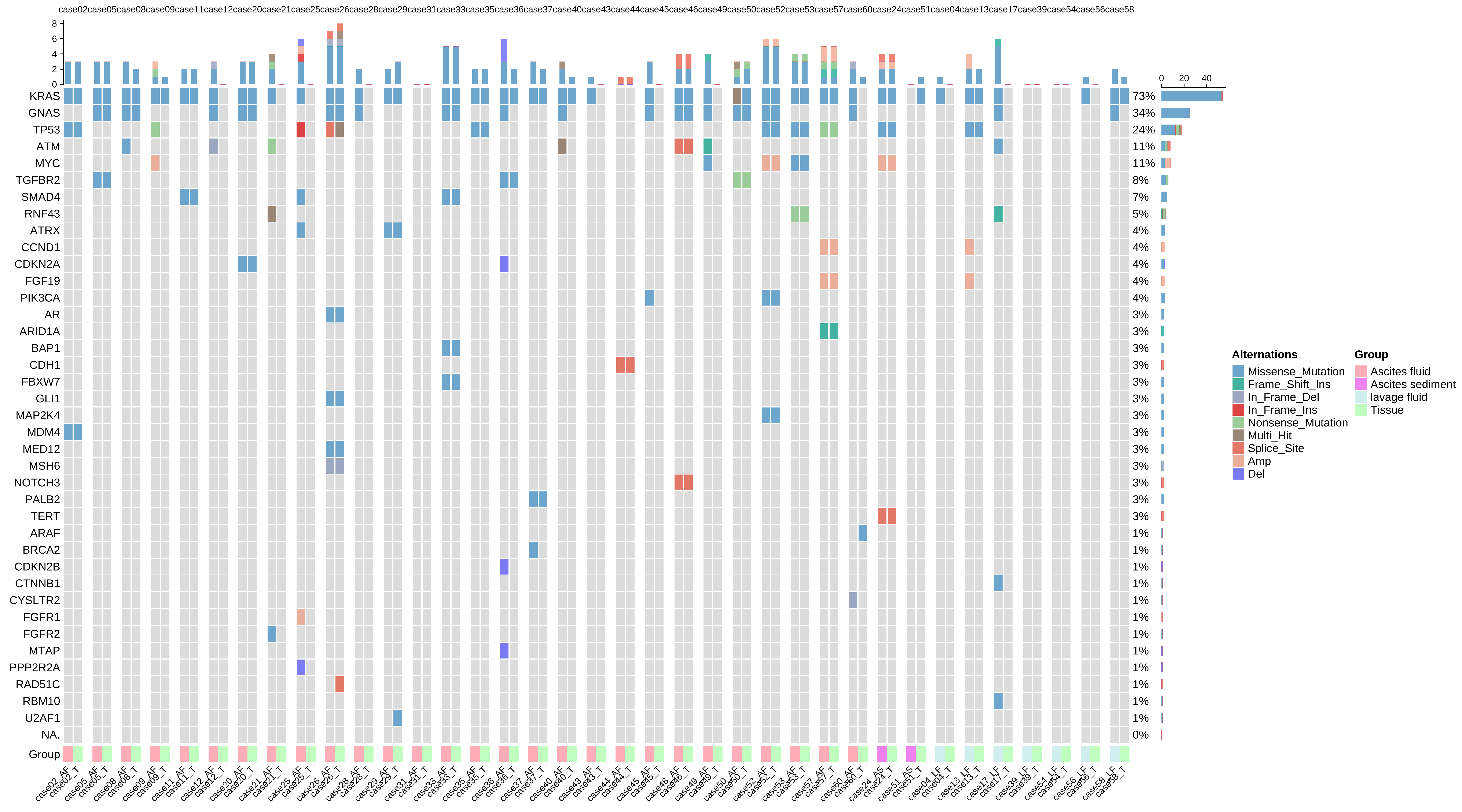
case02 case05 case08 case09 case11 case12 case20 case21 case25 case26 case28 case29 case31 case33 case35 case36 case37 case40 case43 case44 case45 case46 case49 case50 case52 case53 case57 case60 case24 case51 case04 case13 case17 case39 case54 case56 case58
KRAS 73%
GNAS 34%
TP53 24%
ATM 11%
MYC 11%
TGFBR2 8%
SMAD4 7%
RNF43 5%
ATRX 4%
CCND1 4%
CDKN2A 4%
FGF19 4%
PIK3CA 4%
AR 3%
ARID1A 3%
BAP1 3%
CDH1 3%
FBXW7 3%
GLI1 3%
MAP2K4 3%
MDM4 3%
MED12 3%
MSH6 3%
NOTCH3 3%
PALB2 3%
TERT 3%
ARAF 1%
BRCA2 1%
CDKN2B 1%
CTNNB1 1%
CYSLTR2 1%
FGFR1 1%
FGFR2 1%
MTAP 1%
PPP2R2A 1%
RAD51C 1%
RBM10 1%
U2AF1 1%
NA. 0%
Group
Alternations
Missense_Mutation
Frame_Shift_Ins
In_Frame_Del
In_Frame_Ins
Nonsense_Mutation
Multi_Hit
Splice_Site
Amp
Del
Group
Ascites fluid
Ascites sediment
lavage fluid
Tissue
