# Supplementary material for: Multispecimen Genomic Profiling Reveals Inter-Specimen Heterogeneity and Supports Ascites as a Complementary Biospecimen in Pseudomyxoma Peritonei
Source: Diagnostics (Basel). 2026 Oct 23;16(21):3463. doi: 10.3390/diagnostics16213463 (PMC13761630; doi:10.3390/diagnostics16213463)
Supplement: Supplementary file 1 [file diagnostics-16-03463-s001.zip › Figure S3.pdf]

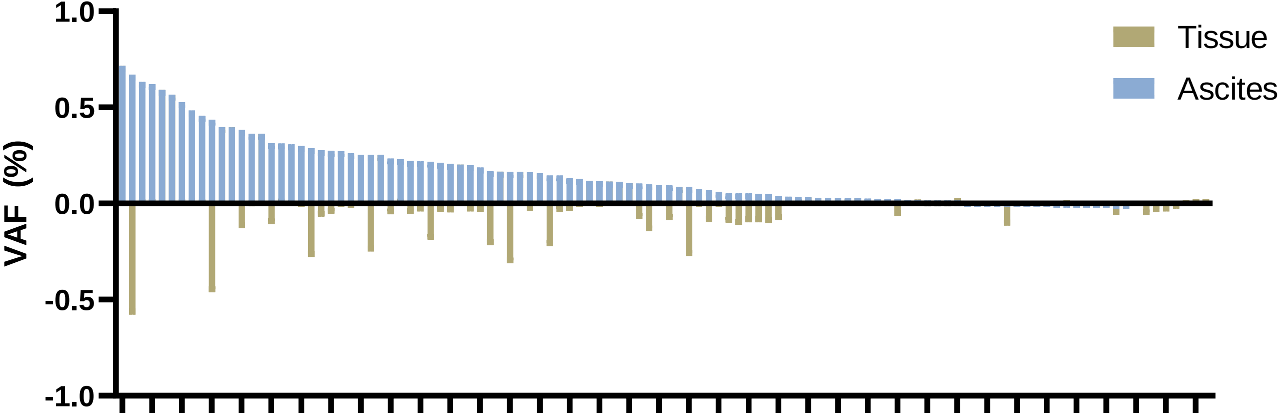
1.0
0.5
0.0
-0.5
-1.0
VAF (%)
Tissue
Ascites
