# Supplementary material for: Multispecimen Genomic Profiling Reveals Inter-Specimen Heterogeneity and Supports Ascites as a Complementary Biospecimen in Pseudomyxoma Peritonei
Source: Diagnostics (Basel). 2026 Oct 23;16(21):3463. doi: 10.3390/diagnostics16213463 (PMC13761630; doi:10.3390/diagnostics16213463)
Supplement: Supplementary file 1 [file diagnostics-16-03463-s001.zip › Figure S4.pdf]

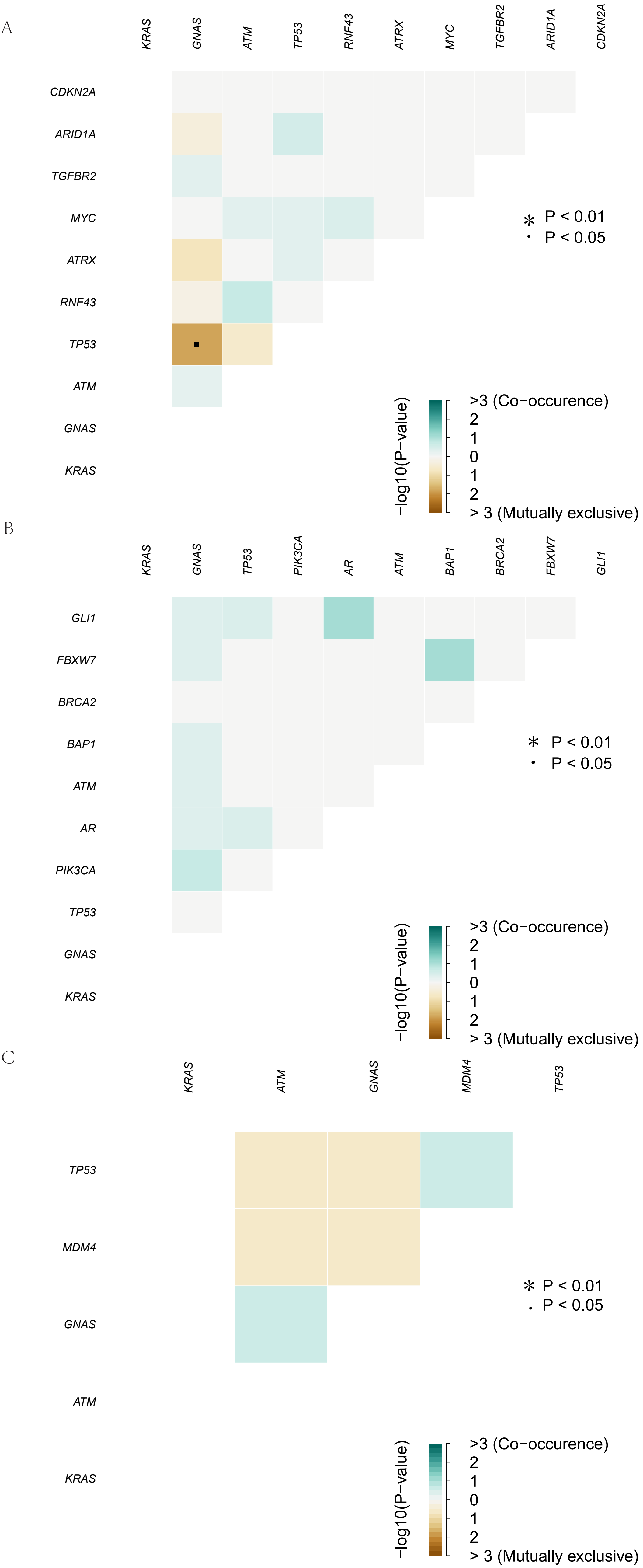
A
KRAS
GNAS
ATM
TP53
RNF43
ATRX
MYC
TGFBR2
ARID1A
CDKN2A
* P < 0.01
· P < 0.05
−log10(P−value)
>3 (Co−occurence)
2
1
0
1
2
> 3 (Mutually exclusive)
B
KRAS
GNAS
TP53
PIK3CA
AR
ATM
BAP1
BRCA2
FBXW7
GLI1
* P < 0.01
· P < 0.05
−log10(P−value)
>3 (Co−occurence)
2
1
0
1
2
> 3 (Mutually exclusive)
C
KRAS
ATM
GNAS
MDM4
TP53
* P < 0.01
· P < 0.05
−log10(P−value)
>3 (Co−occurence)
2
1
0
1
2
> 3 (Mutually exclusive)
